# Gene Expression of Nrf2 and KEAP1 in Monocytes of Patients with Chronic Kidney Disease (CKD)

**DOI:** 10.3390/ijms26199693

**Published:** 2025-10-05

**Authors:** Ahmed Timimi, Subagini Nagarajah, Martin Tepel, Alexandra Scholze

**Affiliations:** 1 Institute of Clinical Research, University of Southern Denmark, 5000 Odense, Denmark; ahtim22@student.sdu.dk (A.T.);; 2 Department of Nephrology, Odense University Hospital, 5000 Odense, Denmark; 3 Institute of Molecular Medicine, Cardiovascular and Renal Research, University of Southern Denmark, 5000 Odense, Denmark; 4 Department of Cardiac, Thoracic, and Vascular Surgery, Odense University Hospital, 5000 Odense, Denmark

**Keywords:** Nrf2, KEAP1, NQO1, CKD, HD, gene expression

## Abstract

In chronic kidney disease (CKD), oxidative stress and inflammation contribute to disease progression and CKD-related morbidity. The nuclear factor erythroid 2-related factor 2 (Nrf2) system plays a central role in the cellular response to oxidative and inflammatory stress. In this brief report, we describe our investigation into whether alterations in the gene expression of key Nrf2 pathway components contribute to the endogenous activation of the Nrf2 system previously reported in less advanced CKD. To this end, we quantified the gene expression of Nrf2, its regulatory protein Kelch-like ECH-associated protein 1 (KEAP1), and the Nrf2 downstream target NAD(P)H:quinone oxidoreductase 1 (NQO1) in monocytes from patients in different stages of CKD. We observed significantly elevated NQO1 gene expression in CKD stage G3b compared to CKD stages G1-3a (*p* < 0.05), G4 (*p* < 0.01), and G5 (*p* < 0.001). In contrast, the gene expression levels of Nrf2 and KEAP1 did not differ significantly between CKD stages. These findings suggest that endogenous activation of the Nrf2 system in moderate CKD predominantly reflects functional activation, likely at the protein level, rather than changes in the gene expression of Nrf2 or KEAP1.

## 1. Introduction

Chronic kidney disease (CKD) is a progressive disease characterized by a gradual decline in kidney function. CKD is a major cause of morbidity and mortality worldwide, affecting more than 10% of the population [1]. A key feature in the pathogenesis of CKD-related morbidity is oxidative stress, which results from an imbalance between prooxidant and antioxidant factors [2] and is evidenced by, e.g., increased reactive oxygen species (ROS) production by mononuclear cells in advanced CKD [3], which contributes to heightened cardiovascular risk in affected patients [4,5]. ROS play an important role in the nuclear factor erythroid 2-related factor (Nrf2) system, which is critical to anti-inflammatory and antioxidative processes [6,7]. Nrf2 acts as gene transcription factor in antioxidant pathways by activating target genes that play a major role in cellular defense against oxidative stress and inflammation. A well-known positive Nrf2 downstream target gene is NAD(P)H:quinone oxidoreductase-1 (NQO1) [8,9].

Under stress-free conditions, cellular Nrf2 protein activity is inhibited by Kelch-like ECH-associated protein (KEAP1), which promotes Nrf2 proteasomal degradation [6,7]. Under oxidative stress, ROS oxidize KEAP1 cysteine residues, modifying the KEAP1 structure and thus preventing the proteasomal degradation of Nrf2. This allows Nrf2 proteins to accumulate and upregulate target genes, such as NQO1.

The Nrf2 system shows typical changes in CKD that depend on the severity of CKD, as well as antioxidant medications, comorbidities, and aging processes [10,11]. Oxidative stress increases with progressing CKD. In advanced stages of CKD, and especially in patients that need hemodialysis therapy, the Nrf2 system is impaired, thereby reinforcing oxidative stress. Nrf2 downregulation in late CKD and hemodialysis was shown in the Nrf2 gene expression level [12,13], Nrf2 protein level [14], and the Nrf2 effector level utilizing the gene expression of Nrf2 downstream target genes [10,15].

On the contrary, until CKD stages G3-4, the Nrf2 system seems to retain its ability to react to oxidative stress [16]. In alignment with this observation, endogenous Nrf2 system activation with upregulated Nrf2 protein concentrations [14] and increased gene expression of Nrf2 target genes [9,16,17,18] was reported for earlier, non-dialysis-dependent CKD stages.

Hence, our study aimed to determine, in monocytes, if regulating the gene expression of Nrf2 system components contributes to endogenous Nrf2 activation in the earlier stages of CKD. We hypothesized that if this was the case, either Nrf2 gene expression would be upregulated or the expression of the Nrf2 inhibitor KEAP1 would be downregulated.

## 2. Results

Table 1 shows the characteristics of the CKD patients included in the study. The population structure, with respect to CKD stage and kidney replacement therapy (KRT), was as follows: CKD G1,2, 2 subjects; CKD G3a, 7 subjects; CKD G3b, 15 subjects; CKD G4, 19 subjects; CKD G5 without KRT, 1 subject; and CKD G5 with hemodialysis therapy, 45 subjects).

**Figure 1:**
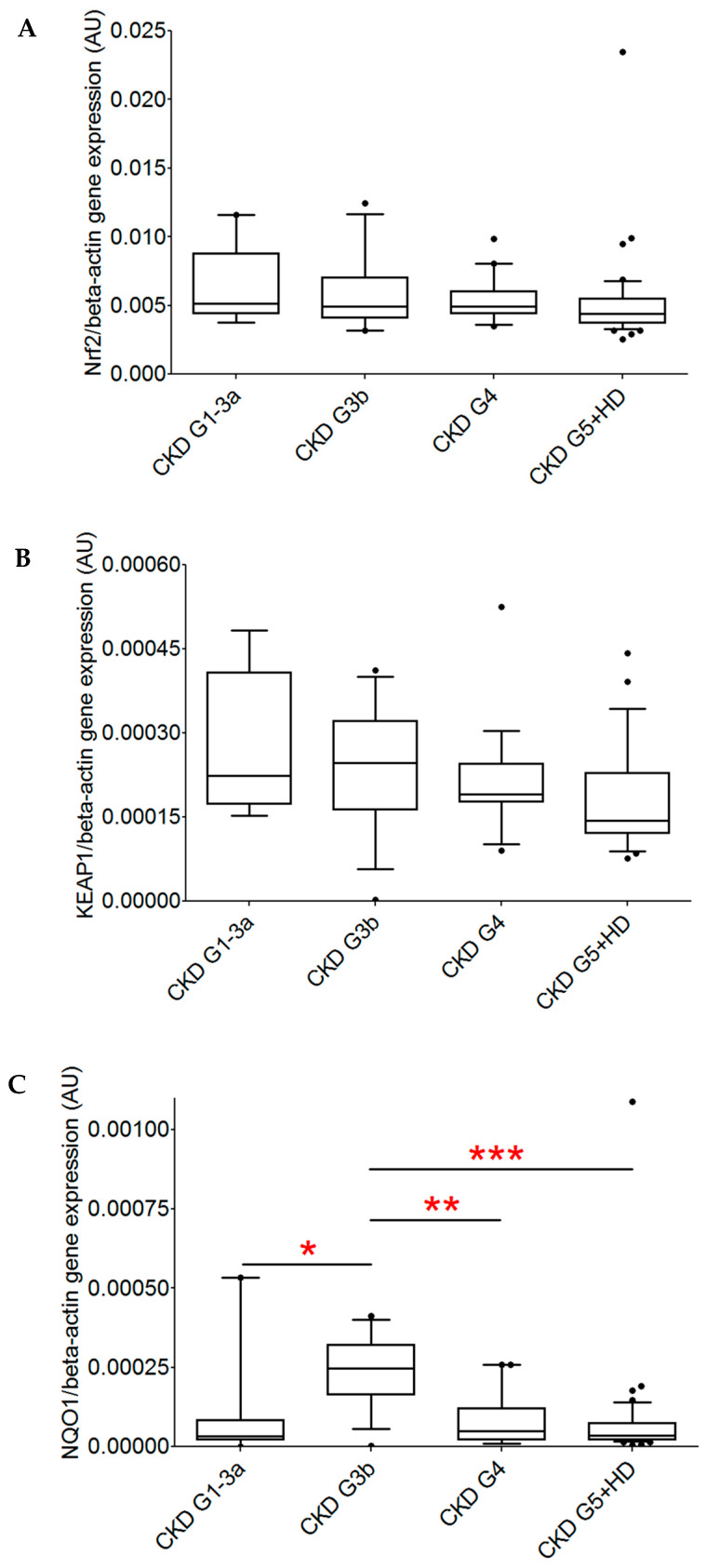
Comparison of normalized gene expression of Nrf2, KEAP1, and NQO1 in patients with CKD. Patients were grouped according to the severity of CKD (CKD G1-3a, n = 9; CKD G3b, n = 15; CKD G4, n = 19; and CKD G5 with and without hemodialysis therapy, n = 46). (**A**) No significant difference in Nrf2 gene expression between the groups was observed. (**B**) No significant differences in KEAP1 gene expression between the groups was observed. (**C**) NQO1 gene expression differed significantly between the groups, with the highest expression observed in CKD G3b (Dunn’s post test: * *p* < 0.05 vs. CKD G1-3a, ** *p* < 0.01 vs. CKD G4, *** *p* < 0.001 vs. CKD G5).

We compared the gene expression of the transcription factor Nrf2; its regulator on the protein level, KEAP1; and the Nrf2 downstream target NQO1 between patients in different stages of CKD. Neither Nrf2 gene expression nor KEAP1 gene expression differed significantly between the groups (Kruskal–Wallis *p* = 0.16 for Nrf2 and 0.05 for KEAP1). On the contrary, gene expression differed significantly between CKD stages for NQO1 (Kruskal–Wallis *p* = 0.0001). As depicted in Figure 1, NQO1 gene expression was the highest in patients with CKD G3b.

Furthermore, we analyzed the relationship of the gene expression of all targets to age and eGFR in patients with CKD who were not on KRT. While Nrf2 and KEAP1 gene expression correlated negatively with age, no such relation was observed for NQO1. No correlation with eGFR was observed for any of the three target genes (Table 2).

## 3. Discussion

This study showed significantly increased gene expression of the Nrf2 downstream target gene NQO1 in monocytes in CKD G3b patients in comparison to patients in CKD stages with only mild kidney impairment but especially compared to patients who had advanced kidney disease and were receiving hemodialysis therapy. This NQO1 upregulation was not explained by upregulated Nrf2 gene expression or downregulated KEAP1 gene expression.

In patients with CKD, oxidative stress contributes to the progression of CKD and to CKD-related morbidity [19,20]. Activation of the Nrf2 system is an adequate reaction to oxidative stress and inflammatory processes. It will induce adaptive responses by upregulating, e.g., NQO1 and thioredoxin gene expression [8]. Our group recently reported upregulated Nrf2 protein concentrations in less advanced CKD compared to advanced CKD [14]. Both the sustained activation of Nrf2 and an inadequate repression of Nrf2 in CKD should be prevented or corrected [7,20]. It is therefore important to understand the mechanisms underlying Nrf2 alterations in CKD. Our analyses did not detect significant differences in the gene expression of Nrf2 or KEAP1 between different CKD stages. Furthermore, in the patient population with CKD, we found no correlation between kidney function, determined using eGFR, and Nrf2 or KEAP1 expression. We observed decreased Nrf2 gene expression with increasing age, which aligns with the literature [21,22].

We confirmed significantly higher expression of the Nrf2 downstream target gene NQO1 in monocytes in patients with moderate–severe impairment of kidney function (CKD3b), which we and others reported earlier [9,16]. Since no upregulation of Nrf2 gene expression or downregulation of KEAP1 gene expression in moderately–severely impaired kidney function was observed in our study, the stimulation of NQO1 gene expression is most likely a physiologic response to the increased oxidative challenge in these patients. ROS will oxidize KEAP1 and thus prevent the proteasomal degradation of Nrf2 protein. This is supported by the increased Nrf2 protein concentrations reported in mild–severe CKD [14]. Furthermore, our study shows that Nrf2 system activity was significantly lower in patients with severe CKD and kidney failure compared to patients with CKD3b, as indicated by low levels of NQO1 gene expression. This can be interpreted as an inadequate antioxidant response and is supported by data from other authors showing a conserved response of NQO1 to electrophiles in non-dialysis CKD stages G3–5 [23] but not in hemodialysis patients [24].

Finally, in addition to the activation of the Nrf2 system on the functional and hence protein level, which includes reduced Nrf2 protein degradation and the resulting regulation of Nrf2 target genes, regulations on the Nrf2 and KEAP1 gene expression levels have been reported. These include the downregulation of Nrf2 by uremic toxins [25] and the upregulation of Nrf2 and KEAP1 gene expression by lipopolysaccharides [26]. Such mechanisms are highly likely in our patient population. This would result in overlapping, opposing effects on Nrf2 and KEAP1 gene expression. The level of Nrf2 gene expression will therefore vary between patients, depending on the strength of suppressing and stimulating factors.

### Limitation

Patients with CKD stages G1 and 2 are only seldom present at the recruiting Department of Nephrology, while patients with severe stages of CKD are seen more frequently. This results in an underrepresentation of patients with G1 and 2 CKD in our study population. Hence, further research needs to confirm our results in a larger CKD population with a more even representation of CKD stages. In addition, our study was performed in monocytes, limiting the generalizability of our results to other cell types without further study.

## 4. Materials and Methods

### 4.1. Study Participants

Consecutive patients with CKD were enrolled from the nephrological outpatient clinic and the hemodialysis unit at the Department of Nephrology, Odense University Hospital, at their regular scheduled visits. The criteria for inclusion and exclusion as specified in the study protocol were applied to all study participants. Inclusion criteria: participant legally competent, >18 years of age, and known with verified CKD. Exclusion criteria: participant pregnant or breastfeeding, with functioning kidney graft, critically ill, or lacking written informed consent.

CKD was defined according to the 2012 Clinical Practice Guideline for the Evaluation and Management of Chronic Kidney Disease [27]. For patients not on hemodialysis therapy, eGFR was determined using the CKD-EPI equation [28] and the stage of CKD was determined based on the respective eGFR [27].

Information on patients’ demographics and medical history, like underlying cause of CKD, was obtained from medical records.

### 4.2. Cell Sample Preparation

Venous blood samples were drawn in the morning from patients with non-dialysis dependent CKD visiting the outpatient clinic. Samples from patients on chronic hemodialysis treatment were obtained before the start of hemodialysis sessions. We isolated peripheral blood mononuclear cells using density gradient centrifugation (Histopaque-1077, Sigma-Aldrich, Søborg, Denmark). CD14-positive monocytes were obtained from these peripheral blood mononuclear cells using anti-CD14-coated superparamagnetic polystyrene particles (Dynabeads; Fisher Scientific, Roskilde, Denmark) [9]. The samples were frozen at −80 °C and stored in a biobank.

### 4.3. RNA Preparation

RNeasy Mini Kit (Qiagen, Denmark) was used according to the manufacturer’s instructions. Biobanked samples were thawed from −80 °C and subsequently mixed with ethanol. A magnet and centrifugation steps allowed separation of the cellular components from the cytosol and the manufacturers protocol (RNeasy Mini Kit) was followed. Finally, the samples were incubated with RNase-free water, centrifuged and frozen at −80 °C. Before further use, RNA concentration of all samples was determined and an optical density 260/280 with a minimal threshold of 1,8 was used to ensure satisfactory levels of purity (IMPLEN photometer, IMPLEN, Munich, Germany).

### 4.4. cDNA Synthesis and Quantitative Real-Time PCR

The cDNA was synthesized from 200 ng of total RNA by reverse transcription (Quantitect Reverse Transcription Kit, Qiagen, Hvidovre, Denmark). The previously isolated RNA samples were thawed and genomic DNA elimination mix was prepared individually for each RNA sample concentration.

Quantitative real-time polymerase chain reaction was performed with a Roche LightCycler^®^ 96 (Roche, Copenhagen, Denmark), a SYBR Green kit (FastStart Essential DNA Green Master, Roche, Denmark) was used after the manufacturer’s instructions. Samples were analyzed in duplicate with the following PCR conditions: 95 °C for 600 s and 50 cycles of 95 °C for 10 s, 64 °C for 10 s, and 72 °C for 10 s. Primers were from Sigma-Aldrich, the primer sequences are given in Table 3.

The delta CT method was used to calculate the relative gene expression level of the targets (Nrf2, KEAP1, NQO1) to the reference gene (ACTB). Beta actin (ACTB) has previously been established by our group as a suitable housekeeping gene with a high gene expression stability in a comparable population of CKD patients [9].

### 4.5. Statistical Analyses

We present categorical variables as absolute numbers and percentages. Distributions of the data were tested by D’Agostino’s and Pearson’s normality tests. Continuous variables are reported as medians and interquartile ranges (IQR), and comparisons were performed using Kruskal–Wallis with post hoc Dunn’s test. Non-parametric bivariate Spearman correlation analysis was performed. We used GraphPad prism software, version 5.0 (GraphPad Software, San Diego, CA, USA), and a two-sided threshold of *p* < 0.05 was used for statistical significance.

## Figures and Tables

**Table 1:** The clinical and biochemical data of patients with CKD included in this study.

	CKD G1–G5 (n = 89)
**Age, years**	67 (55–75)
**Men, n (%)**	53 (60)
**Kidney disease, underlying cause, n (%)**	
** Glomerulonephritis**	14 (16)
** Diabetic nephropathy**	6 (7)
** Hypertensive nephropathy**	6 (7)
** Other/unknown**	63 (71)
**eGFR, mL/min/1.73 m^2^**	30 (24.3–40.8) *
**Hemodialysis therapy**	45 (51)

Data are given as median (25–75% percentile) values and numbers (percentages), as appropriate. CKD G1–G5, chronic kidney disease eGFR stages 1–5; HD, hemodialysis therapy; eGFR, estimated glomerular filtration rate. * data from all patients without hemodialysis therapy (n = 43).

**Table 2:** Correlation analysis for Nrf2, KEAP1, and NQO1 gene expression in patients with CKD not treated with hemodialysis (n = 43).

	Nrf2 Gene Expression		KEAP1 Gene Expression		NQO1 Gene Expression	
	r	*p*	r	*p*	r	*p*
Age	−0.3	0.04	−0.3	0.04	−0.12	0.43
eGFR, mL/min/1.73 m^2^	0.04	0.79	0.17	0.28	−0.03	0.85

Nrf2, nuclear factor erythroid 2-related factor 2; KEAP1, Kelch-like ECH-associated protein 1; NQO1, NAD(P)H: quinone oxidoreductase 1; eGFR, estimated glomerular filtration rate; r, Spearman correlation coefficient.

**Table 3:** Primer sequences.

Study Target (Gene Name)	Gene Accession Numbers	Forward Primer Reverse Primer
Nrf2 (*NFE2L2*)	NM_006164	5′-TTCAGCCAGCCCAGCACATC-3′ 5′-CGTAGCCGAAGAAACCTCATTGTC-3′
KEAP1 (*Keap1*)	NM_203500	5′-GGGTCCCCTACAGCCAAG-3′ 5′-TGGGGTTCCAGAAGATAA GC-3′
NQO1 (*NQO1*)	NM_000903.2	5′-CTGCCATCATGCCTGACTAA-3′ 5′-TGCAGATGTACGGTGTGGAT-3′
ACTB (*ACTB*)	NM_001101.3	5′-GGACTTCGAGCAAGAGATGG-3′ 5′-AGCACTGTGTTGGCGTACAG-3′

Nrf2, nuclear factor erythroid 2-related factor 2; KEAP1, Kelch-like ECH-associated protein 1; NQO1, NAD(P)H: quinone oxidoreductase 1; ACTB, Beta actin.

## Data Availability

Due to ethical and data privacy restrictions, patient-related data cannot be made available.

## Abbreviations

The following abbreviations are used in this manuscript:

ACTB	Beta actin
CKD G1–G5	Chronic kidney disease eGFR stages 1–5
eGFR	Estimated glomerular filtration rate
HD	Hemodialysis therapy
KEAP1	Kelch-like ECH-associated protein 1
KRT	Kidney replacement therapy
NQO1	NAD(P)H:quinone oxidoreductase 1
Nrf2	Nuclear factor erythroid 2-related factor
ROS	Reactive oxygen species
